# Pyrosequencing the transcriptome of the greenhouse whitefly, *Trialeurodes vaporariorum *reveals multiple transcripts encoding insecticide targets and detoxifying enzymes

**DOI:** 10.1186/1471-2164-12-56

**Published:** 2011-01-24

**Authors:** Nikos Karatolos, Yannick Pauchet, Paul Wilkinson, Ritika Chauhan, Ian Denholm, Kevin Gorman, David R Nelson, Chris Bass, Richard H ffrench-Constant, Martin S Williamson

**Affiliations:** 1 Rothamsted Research, Harpenden, Hertfordshire, AL5 2JQ, UK; 2 Biosciences, University of Exeter, Penryn, TR10 9EZ, UK; 3 Max Planck Institute for Chemical Ecology, 07745 Jena, Germany; 4 Molecular Science, University of Tennessee, Memphis, USA

## Abstract

**Background:**

The whitefly *Trialeurodes vaporariorum *is an economically important crop pest in temperate regions that has developed resistance to most classes of insecticides. However, the molecular mechanisms underlying resistance have not been characterised and, to date, progress has been hampered by a lack of nucleotide sequence data for this species. Here, we use pyrosequencing on the Roche 454-FLX platform to produce a substantial and annotated EST dataset. This 'unigene set' will form a critical reference point for quantitation of over-expressed messages via digital transcriptomics.

**Results:**

Pyrosequencing produced around a million sequencing reads that assembled into 54,748 contigs, with an average length of 965 bp, representing a dramatic expansion of existing cDNA sequences available for *T. vaporariorum *(only 43 entries in GenBank at the time of this publication). BLAST searching of non-redundant databases returned 20,333 significant matches and those gene families potentially encoding gene products involved in insecticide resistance were manually curated and annotated. These include, enzymes potentially involved in the detoxification of xenobiotics and those encoding the targets of the major chemical classes of insecticides. A total of 57 P450s, 17 GSTs and 27 CCEs were identified along with 30 contigs encoding the target proteins of six different insecticide classes.

**Conclusion:**

Here, we have developed new transcriptomic resources for *T. vaporariorum*. These include a substantial and annotated EST dataset that will serve the community studying this important crop pest and will elucidate further the molecular mechanisms underlying insecticide resistance.

## Background

Whiteflies (Hemiptera: Aleyrodidae) are important pests of agriculture that feed on and transmit viruses to a wide range of crops. The two most damaging and widespread species are the tobacco or cotton whitefly (*Bemisia tabaci *Gennadius) and the greenhouse whitefly (*Trialeurodes vaporariorum *Westwood).

One factor enhancing the pest status of whiteflies is their ability to evolve resistance to insecticides. Both *B. tabaci *and *T. vaporariorum *are known to exhibit resistance to several insecticide groups including the neonicotinoids, the most widely-used compounds for whitefly control [1,2]. Insecticide resistance commonly arises through two main mechanisms 1) reduced binding of the insecticide to its target through target site mutation [3] (e.g. acetylcholinesterase for organophosphates/carbamates, the voltage-gated sodium channel for pyrethroids) and 2) enhanced metabolism or sequestration of insecticide by enzymes such as carboxyl-cholinesterases (CCEs), glutathione-S-transferases (GSTs) and cytochrome P450 monooxygenases [3-7].

CCEs, GSTs and P450s are encoded by large and diverse gene families that are difficult to fully characterise by traditional biochemical methods. Identification and cloning of genes encoding insecticide target sites composed of multiple subunit proteins (such as the nicotinic acetylcholine receptor) by degenerate PCR is also a lengthy and sometimes difficult process. The recent and rapid growth of the use of next generation sequencing has made it easier to study large complex genes or gene families such as insecticide target sites and those involved in detoxification of xenobiotics via the de novo sequencing of whole insect transcriptomes [8,9]. Although there is a significant amount of genomic data for *B. tabaci *in this regard, including an expressed sequence tag (EST) library [10] and an ongoing genome project [11], very little comparable data for *T. vaporariorum *exist, with only 43 nucleotide sequences currently available at NCBI.

Cost-effective high-throughput DNA sequencing technologies such as 454-based pyrosequencing of ESTs are a powerful new approach to characterise the transcriptome of insect species that lack a fully sequenced genome [8,9,12]. The amount of sequence information generated by these methods also facilitates the global analysis of gene expression by providing a reference transcriptome for cDNA microarray design and/or Serial Analysis of Gene Expression (SAGE) [13,14,10]. Here, we have used 454-based pyrosequencing to generate a substantial EST dataset of the *T. vaporariorum *transcriptome and then characterised genes encoding detoxification enzymes and insecticide target proteins.

## Results and discussion

### 454 pyrosequencing and assembly

**Figure 1:**
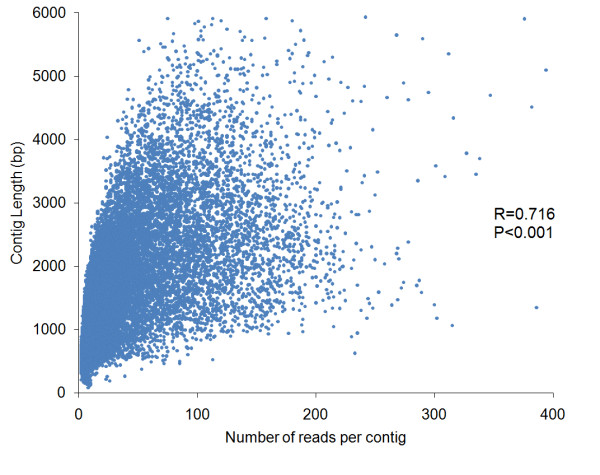
**Scatter plot of number of reads representing a contig versus the contig length**. Summary of correlation statistics is shown.

Over-abundant 0.6-6 kb transcripts were reduced by normalisation of the whitefly cDNAs and an even distribution of transcripts ranging from 0.5 to 6 kb in size was produced. 454 pyrosequencing of two libraries (from the insecticide-susceptible TV1 and the imidacloprid selected TV6 *T. vaporariorum *strains) resulted in a total of 1,104,651 reads. After quality scoring of the reads, 990,945 high-quality reads with an average length of 362 bp were entered to assembly (est2assembly). One pooled assembly was done incorporating both libraries (52,832,938 bp of sequencing), which resulted in 54,748 contigs with an average length of 965 bp (Table 1). 55.8% (30,552 contigs) of these had an ORF (open reading frame) ≥200 bp, with an average length of 540 bp. The characteristics of the assembled *T. vaporariorum *454 contigs and BLASTx alignments against the *Drosophila melanogaster *uniprot database are shown in Additional file 1. Figure 1 demonstrates that contigs that were assembled from up to 200 reads displayed a linear relationship between sequence read number and contig length (R = 0.716, P < 0.001).

**Table 1:** Summary statistics for *Trialeurodes vaporariorum *EST assembly and annotation

Assembly	
Total number of reads	1,104,651
Number of reads after pre-processing	990,945
Average read length after pre-processing	362 bp
Total number of contigs	54,748
Average contig length	965 bp
Sequencing length	52,832,938 bp
Contigs with ORF ≥200 bp (average length)	30,552 (540 bp)
Average read coverage per contig	4.34x
Average GC % content of contigs	37.77%
**Annotation**	
% contigs with at least 1 GO term	30.01%
% contigs with an EC number	6.10%
% contigs with at least 1 IPR	33.27%

**Contigs with at least 1 blast hit against nr**	
Total number	20,333
Average length	1,394 bp
% of those contigs with at least 1 IPR	42.61%
% of those contigs with at least 1 GO term	78.43%
Contigs with ORF ≥200 bp (average length)	18,080 (704 bp)

**Contigs with no blast hits**	
Total number	34,416
Average length	712 bp
% of those contigs with at least 1 GO term	1.41%
% of those contigs with at least 1 IPR	28.01%
Contigs with ORF ≥200 bp (average length)	12,472 (301 bp)

* nr: non-redundant database, GO: gene ontology term, EC: enzyme commission number, IPR: inter-pro result, ORF: open reading frame.

### Homology searches, gene ontology and protein classification

**Figure 2:**
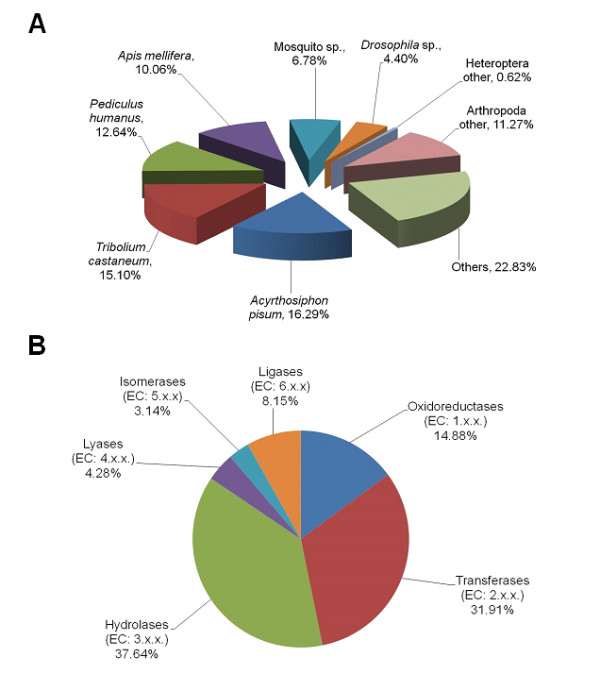
**Species distribution of the top BLAST hit in the nr database for each contig of *Trialeurodes vaporariorum *(A) and general Enzyme Classification (EC) terms for the contigs of *T. vaporariorum *(B)**.

Approximately 37% (20,333 sequences) of the contigs returned an above cut-off BLAST hit to the NCBI nr database (1E^-3 ^for BLASTx resulted in 19,983 and 1E^-10 ^for BLASTn resulted in 350 additional BLAST results) (Additional file 2). The average read length of these contigs was 1,394 bp and E-value and sequence similarity distributions are detailed in Additional file 3. As expected, the pea aphid *Acyrthosiphon pisum *Harris (Hemiptera: Aphididae) is the species that returned the most BLAST hits (16%) with the *T. vaporariorum *contigs (Figure 2A), since this species' genome was recently fully sequenced [15] and currently represents the vast majority of hemipteran sequences available in GenBank.

The remaining 34,416 contigs that did not return a significant BLAST result against the NCBI nr database, had an average read length of 712 bp. More than 36% of those contigs (12,472 sequences) were found to have an ORF ≥200 bp, with an average length of 301 bp. 28% of these contigs (9,553 sequences) returned an InterPro result and 1.4% (488 sequences) returned a GO term (Table 1). These results give some indication of the limitation of BLAST comparison as a tool for inferring the relevant biological function of tentative unique genes assembled from sequencing data for species with very limited existing transcriptomic information. However, it is likely that the rapid expansion in sequence data from ongoing small and large scale insect sequencing projects will facilitate the future annotation of these genes.

**Figure 3:**
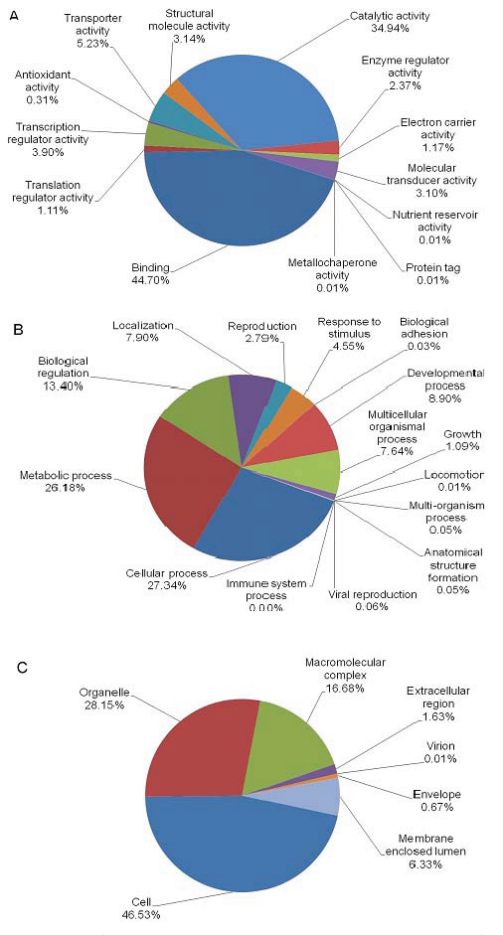
**Gene ontology (GO) assignments for the *Trialeurodes vaporariorum *transcriptome**. A. Molecular function GO terms, B. Biological process GO terms, C. Cellular component GO terms. The data presented represent the level 2 analysis, illustrating general functional categories.

GO terms were used for the classification of the functions of the predicted whitefly proteins, producing 21,899 terms for biological process categories, 15,571 for molecular function categories, and 14,966 for cellular component categories. Enzyme classification shows that hydrolases account for the largest proportion of *T. vaporariorum *enzymes (38%), followed by transferases (32%) and oxidoreductases (15%) (Figure 2B). Most of the molecular function GO terms (Figure 3A) were involved in binding (45%) followed by catalytic activity (35%). Metabolic and cellular processes were involved with more than a half of the biological process GO terms (Figure 3B). The overall distribution suggests that the sequencing provided a comprehensive representation of the *T. vaporariorum *transcriptome and that 454 pyrosequencing of ESTs can achieve a great number and depth of sequence contigs.

### Transcripts encoding genes involved in insecticide detoxification

*Trialeurodes vaporariorum*, like most insect species, metabolises xenobiotics such as secondary plant chemicals and insecticides using a suite of detoxification enzymes such as P450s, GSTs and CCEs. Representatives of all three enzyme families were identified in the *T. vaporariorum *transcriptome and the average sequence length and coverage obtained for members of these gene families using one full plate of 454 sequencing was comprehensive (Table 2). Table 2 reveals that the average ORF length of each of these gene families obtained for *T. vaporariorum *was more than 84% of that of the same gene families in the fully-annotated genome of *A. pisum*. Candidate contigs were manually curated to identify allelic variants of the same gene or those with a high number of sequencing errors. Most contigs were assembled from at least 4 sequencing reads and were identified in both *T. vaporariorum *libraries (TV1 and TV6). The only exception was contig 12863, which was assembled from 19 reads from only the imidacloprid resistant strain (TV6) library and had a protein motif of a CCE.

**Table 2:** Summary information for the identified cytochrome P450s, carboxyl/cholinesterases (CCEs) and glutathione-S transferases (GSTs) in the *Trialeurodes vaporariorum *transcriptome

					Average ORF length	
					
Genes	Number of identified contigs	Average contig size	Average reads per contig	Average coverage per contig	***Trialeurodes vaporariorum***^**1**^	***Acyrthosiphon pisum***^**2**^
P450s	123	1,532 bp	37	6.3x	1,442 bp	1,508 bp
CCEs	78	1,394 bp	27	4.9x	1,487 bp	1,768 bp
GSTs	44	1,111 bp	44	11.3x	672 bp	756 bp

^1 ^The average ORF length of all the manually curated genes belonging to a certain family identified in *Trialeurodes vaporariorum*.

^2 ^The average ORF length of the full-length genes belonging to a certain family identified in the fully-annotated genome of *Acyrthosiphon pisum*. The sequences were taken from http://www.aphidbase.com/aphidbase.

### Transcripts encoding putative P450s

**Figure 4:**
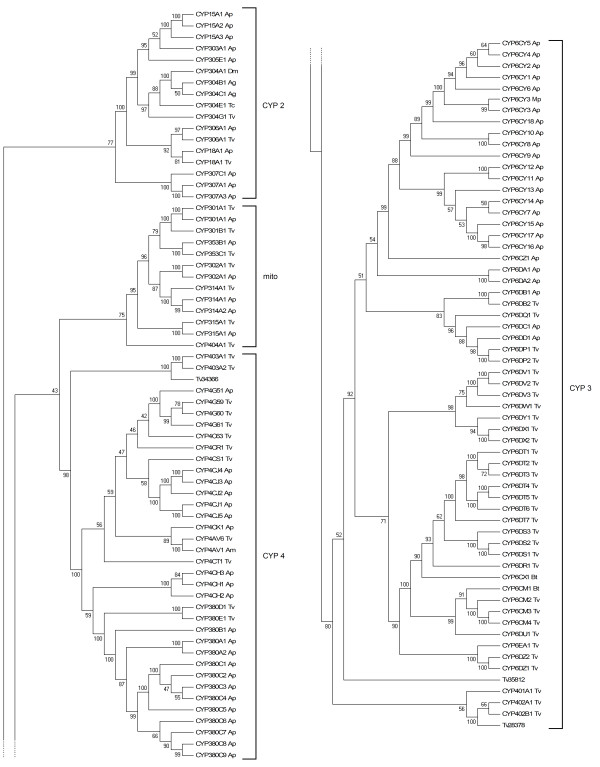
**Neighbour-joining phylogenetic analysis of cytochrome P450s from *Trialeurodes vaporariorum *(Tv) and other insect species**. Bootstrap values next to the nodes represent the percentage of 1000 replicate trees that preserved the corresponding clade. Positions containing alignment gaps and missing data were eliminated with pairwise deletion. *Acyrthosiphon pisum *(Ap), *Bemisia tabaci *(Bt), *Myzus persicae *(Mp), *Drosophila melanogaster *(Dm), *Anopheles gambiae *(Ag), *Tribolium castaneum *(Tc) and *Apis mellifera *(Am) sequences were taken from http://drnelson.uthsc.edu/aphid.htm[16].

A total of 123 P450 related contigs were identified in the transcriptome. Of these, 57 were manually curated (Additional file 4 and Additional file 5) as the remainder were found to be either allelic variants of the same P450 gene or contained too many sequencing errors. These 57 P450 sequences were named by Dr David Nelson in accordance with the P450 nomenclature committee convention (http://drnelson.uthsc.edu/cytochromeP450.html) [16] and 40 of them were found to represent full length ORFs. Based on the closest BLAST hits in the NCBI nr database, and when possible, by phylogenetic analyses with other known insect P450 genes, P450s were assigned to appropriate CYP clades and families. Representatives of all 4 major insect CYP clades (CYP2-4 and mitochondrial) were found in this dataset (Figure 4). A majority of identified P450s belonged to the CYP3 family (34/57 P450s), 13 to the CYP4 family, and the rest to the CYP2 and mitochondrial families (3 and 7 respectively) (Table 3). Phylogenetic analysis of the *T. vaporariorum *P450s with those of *A. pisum *(Figure 4) revealed significant divergence in this gene family between these two species with only a few putative *A. pisum *orthologues identified (Additional file 4). Two sequences were considered orthologues if they were paired in the phylogeny with bootstrap support greater than 50%. Duplication events specific to *T. vaporariorum *are also apparent from the phylogeny, with the best example being the three CYP4-type sequences *CYP4G59*, *CYP4G60 *and *CYP4G61*. The potential role of these duplication events in insecticide resistance warrants further investigation as amplification of a P450 gene has recently been implicated in insecticide resistance in *Myzus persicae *Sulzer (Hemiptera: Aphididae) [13].

**Table 3:** Number of validated GSTs, CCEs and cytochrome P450s annotated in *Trialeurodes vaporariorum *(this study), *Acyrthosiphon pisum*, *Myzus persicae *[23] and *Apis mellifera *[21] genomes and their distribution across classes and clades

	Gene numbers			
Enzymes/Class	*Trialeurodes vaporariorum*	*Acyrthosiphon pisum*	*Myzus persicae*	*Apis mellifera*
**Cytochrome P450s**				
CYP2	3	10	3	8
CYP3	34	33	63	28
CYP4	13	32	48	4
Mitochondrial P450s	7	8	1	6
**Total P450s**	**57**	**83**	**115**	**46**

**Carboxyl/cholinesterases**				
Dietary class				
A clade	11	5	5	8
B clade	0	0	0	0
C clade	1	0	0	0
Hormone/semiochemical processing				
D clade	0	0	0	1
E clade	6	18	12	3
F clade	0	0	0	0
G clade	0	0	0	1
Neurodevelopmental				
H clade	1	1	0	0
I clade	1	0	1	2
J clade	2	2	3	2
K clade	1	1	1	1
L clade	3	3	0	5
M clade	1	0	0	1
**Total CCEs**	**27**	**29**	**22**	**24**

**Cytosolic GSTs**				
Delta	9	10	8	1
Epsilon	1	0	0	0
Omega	0	0	0	1
Sigma	5	6	8	4
Theta	0	2	2	1
Zeta	1	0	0	1
Microsomal	1	2	2	2
**Total cytosolic GSTs**	**17**	**20**	**21**	**10**

CYP3 and CYP4 P450 families in other insect species are implicated in the metabolism of plant secondary metabolites and synthetic insecticides [5]. In the other hemipterans *B. tabaci *and *M. persicae*, over-expression of cytochrome P450s (*CYP6CM1 *and *CYP6CY3 *respectively) contribute to resistance to neonicotinoid insecticides [17,13]. The closest hits of these two P450s in *T. vaporariorum *are *CYP6CM2*, *CYP6CM3 *(68% and 67% similarity to *CYP6CM1 *respectively) and *CYP6DP1, CYP6DZ1 *(60% and 59% similarity to *CYP6CY3 *respectively). These genes and the other CYP3 and CYP4 P450 genes identified in this study are candidates for a potential role in neonicotinoid resistance in *T. vaporariorum*.

Although the number of P450s in the *T. vaporariorum *transcriptome (57) is within the range of P450s identified in other insect species (46-164) [18], additional P450 genes may await discovery due to their absence from the current transcriptomic dataset. Analysis of fully sequenced insect genomes have identified 164 P450s in *Aedes aegypti *Linnaeus (Diptera: Culicidae), 106 in *Anopheles gambiae *Giles (Diptera: Culicidae), 85 in *Drosophila melanogaster *Meigen (Diptera: Drosophilidae), 115 in the green peach aphid *M. persicae*, 83 in the green pea aphid *A. pisum*, and 46 in the western honey bee *Apis mellifera *Linnaeus (Hymenoptera: Apidae) [18-23]. The current number of 57 P450s in *T. vaporariorum *is at the lower end of this range, almost half of that for *M. persicae*.

### Transcripts encoding putative CCEs

**Figure 5:**
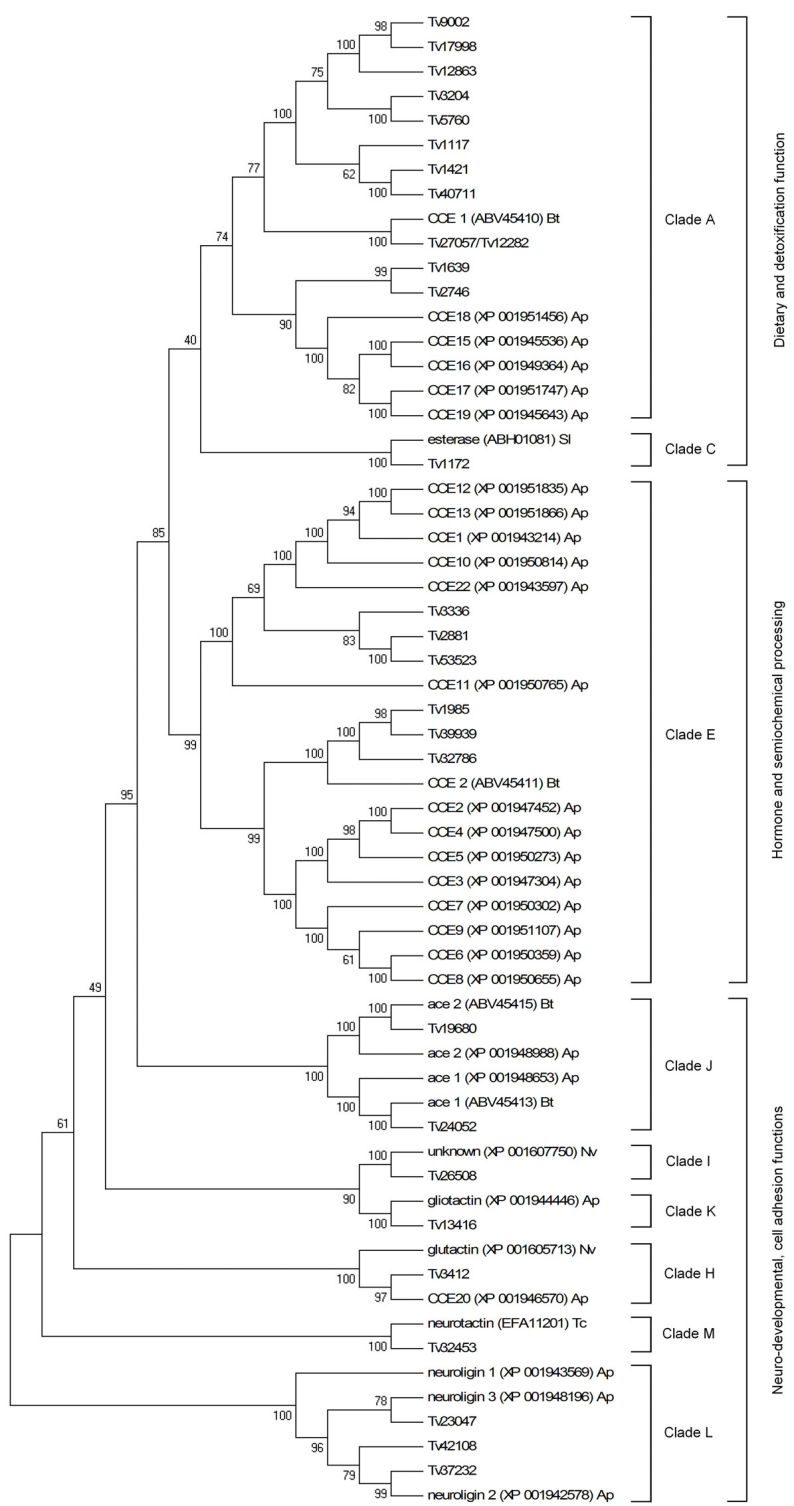
**Neighbour-joining phylogenetic analysis of carboxyl/cholinesterases from *Trialeurodes vaporariorum *(Tv) and other insect species (accession numbers are given)**. Bootstrap values next to the nodes represent the percentage of 1000 replicate trees that preserved the corresponding clade. Positions containing alignment gaps and missing data were eliminated only with pairwise deletion. *Acyrthosiphon pisum *(Ap), *Bemisia tabaci *(Bt), *Nasonia vitripennis *(Nv), *Spodoptera littoralis *(Sl), *Tribolium castaneum *(Tc). *Acyrthosiphon pisum *sequences were taken from http://www.aphidbase.com/aphidbase.

A total of 78 contig sequences with a protein motif of a CCE were identified. Of these, 27 were manually curated (Additional file 4 and Additional file 6) as some of the original sequences were found to be either allelic variants of the same CCE gene or contained too many sequencing errors, and 14 were found to be full length. Based on the closest BLAST hits in the NCBI nr database and when possible by phylogenetic analyses with other known CCE genes from other insect species, these enzymes were assigned to three known classes of CCEs (Figure 5; Table 3). Known CCEs can be divided into 13 clades, nine of which are represented in *T. vaporariorum*. Clades without identifiable *T. vaporariorum *homologues are clade B (alpha esterase), integument esterases (D) and lepidopteran juvenile hormone esterase (F and G). Esterases involved in the detoxification of insecticides belong to clades A-C and 12 sequences were assigned to these clades. A phylogenetic analysis indicates a potential expansion of *T. vaporariorum *CCEs in clade A (compared to known, aphid CCEs). There is also evidence of a contraction in clade E, which contains the vast majority of aphid CCEs, although it is difficult to ascertain if genes in this clade have been lost from the genome or simply remain to be discovered (Figure 5; Table 3). In addition, divergence in the CCEs of *A. pisum *and *T. vaporariorum *is apparent in the same clades (Figure 5). The potential *A. pisum *and *B. tabaci *orthologues of *T. vaporariorum *CCEs are detailed in Additional file 4.

Clade A contains the largest number of identified *T. vaporariorum *CCEs (11 sequences), twice as many as in two other hemipteran species *A. pisum *and *M. persicae *(5 sequences each) [23]. Of these, one CCE sequence (contig 12282) had a high homology to a carboxylesterase gene in *B. tabaci *(COE1; accession ABV45410), which is over-expressed in organophosphate-resistant strains [25] and another (contig 12863) was identified only in the imidacloprid resistant TV6 library and is therefore a candidate gene for a potential role in the neonicotinoid resistance of this strain. One identified sequence (contig 1172) had high homology to Lepidoptera-specific alpha esterase (C). Six sequences had homology to beta esterase (E) and two contigs were identified as acetylcholinesterases (AChE, clade J), which are the targets for organophosphate and carbamate insecticides. One of these, contig 19680, corresponds to a known AChE sequence of *T. vaporariorum *(*ace-2*; accession number CAE11223). Finally, other clades with identified *T. vaporariorum *homologues are glutactin (H), gliotactin (K), neuroligin (L), neurotactin (M) and an uncharacterised group (I).

### Transcripts encoding putative GSTs

**Figure 6:**
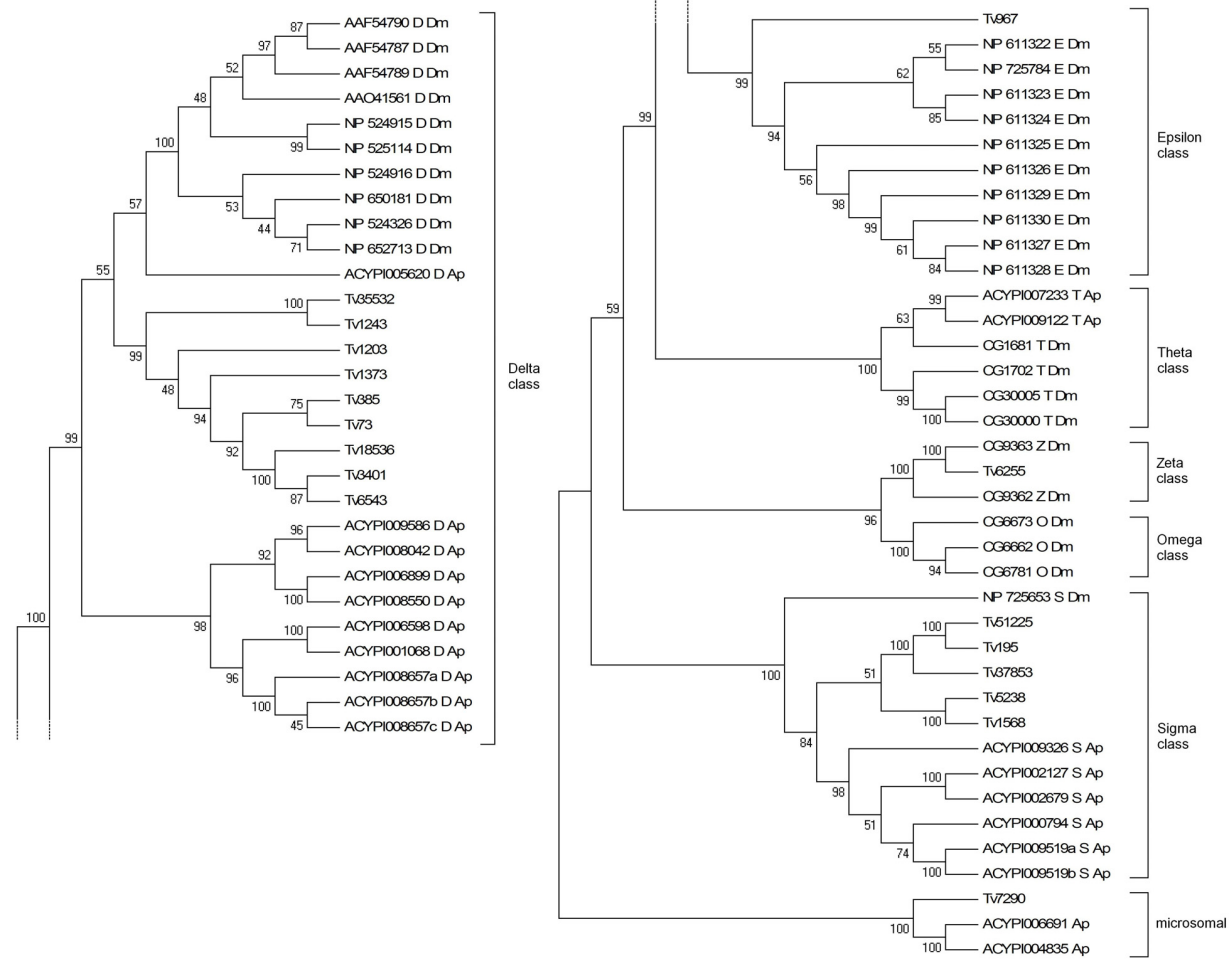
**Neighbour-joining phylogenetic analysis of glutathione-S-transferases from *Trialeurodes vaporariorum *(Tv) and other insect species (accession numbers are given)**. Bootstrap values next to the nodes represent the percentage of 1000 replicate trees that preserved the corresponding clade. Positions containing alignment gaps and missing data were eliminated only with pairwise deletion. *Acyrthosiphon pisum *(Ap), *Drosophila melanogaster *(Dm). *Acyrthosiphon pisum *sequences were taken from http://www.aphidbase.com/aphidbase.

A total of 44 GST-related contig sequences were identified, 17 of which were unique and manually curated (Additional file 4 and Additional file 7), and thirteen of these were full length. Based on the closest BLAST hits in the NCBI nr database and when possible by phylogenetic analysis these contigs were assigned to the Delta, Epsilon, Omega, Sigma, Theta, Zeta, and microsomal classes (Figure 6; Table 3). Phylogenetic comparison of *A. pisum *and *T. vaporariorum *GSTs revealed significant divergence in this gene family between the two species (Figure 6). Most of the identified GSTs were assigned to the Delta class (9 sequences), members of which are known to play a role in insecticide detoxification in other insect species [21]. The number of Delta class GSTs in *T. vaporariorum *(9 sequences) is close to that in *A. pisum *where 10 sequences were identified [23]. Although the Epsilon and Zeta classes are absent in *A. pisum *and *M. persicae *[23], two contigs (one for each class) were identified in *T. vaporariorum*. One contig, namely Tv7290, was found to encode a microsomal GST.

### Detection of gene sequences encoding insecticide targets

**Figure 7:**
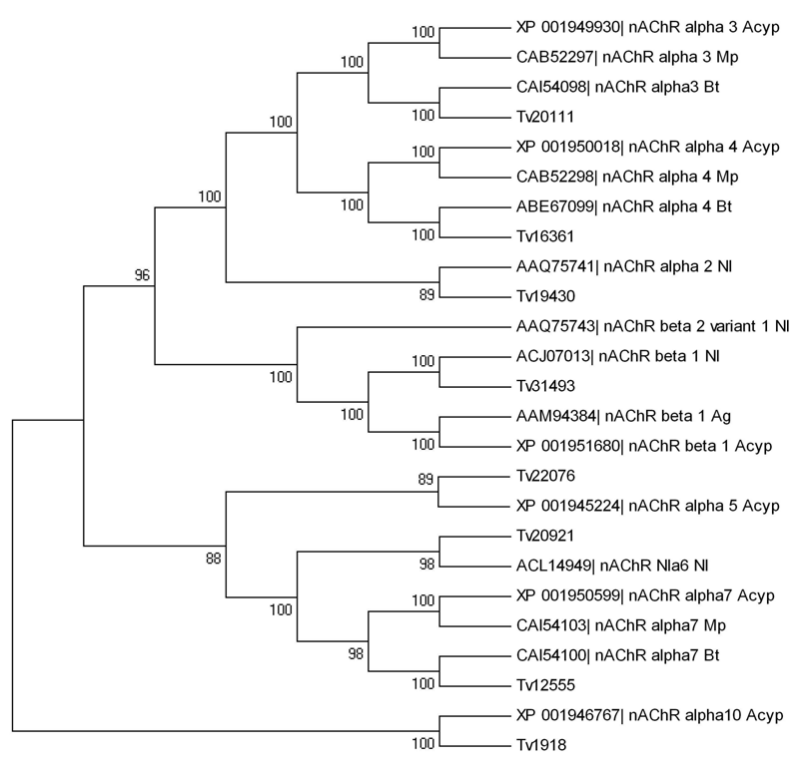
**Neighbour-joining phylogenetic analysis of nicotinic acetylcholine receptors (nAChR) from *Trialeurodes vaporariorum *(Tv) and other insect species (accession numbers are given)**. Bootstrap values next to the nodes represent the percentage of 1000 replicate trees that preserved the corresponding clade. Positions containing alignment gaps and missing data were eliminated only with pairwise deletion. *Acyrthosiphon pisum *(Acyp), *Myzus persicae *(Mp), *Bemisia tabaci *(Bt), *Nilaparvata lugens *(Nl) and *Aphis gossypii *(Ag).

A number of contigs encoding insecticide target proteins were identified in the *T. vaporariorum *transcriptome. These include the acetylcholinesterase enzyme (AChE), nicotinic acetylcholine receptor subunits (nAChRs), the acetyl-CoA carboxylase (ACCase), the voltage-gated sodium channel (VGSC), the γ-aminobutyric acid (GABA) receptor, the glutamate-gated chloride channel (GluCl) and the ryanodine receptor (RyR) (Table 4, Figure 7 and Additional file 8). All contigs that were assembled from more than 3 reads and the vast majority of the contigs with lower coverage were identified in both *T. vaporariorum *libraries (TV1 and TV6). Although many of these contigs are not full length, they will nevertheless facilitate further characterisation of these targets by PCR and/or RACE. As the two cDNA libraries were tagged prior to sequencing, we investigated the occurrence of SNPs in contigs encoding insecticide target-sites between the resistant and susceptible *T. vaporariorum *strains. A limited number of non-synonymous SNPs were observed between the two strains and these are listed in Additional file 9. A number of, often highly conserved, mutations have been described in many of these target proteins that lead to varying degrees of insensitivity such as mutations within the active-site gorge of the AChE enzyme [26], in domains II or III of the VGSC [27], in the pore lining M2 region of the GABA receptor [28] and within two alpha subunits of the nAChR [29]. Where possible we examined the two *T. vaporariorum *libraries for previously described mutations at known 'hot-spots' in other arthropod species but none were observed. However, several non-synonymous mutations at alternative positions in many of the target site genes were found (Additional file 9) and these clearly warrant further investigation. Pyrosequencing or TaqMan^® ^assays can now be rapidly developed and used to screen additional whitefly populations with different resistance phenotypes to determine the consistency of the correlation of these SNPs with resistance.

**Table 4:** Validated genes related to insecticide target sites in *Trialeurodes vaporariorum*.

Insecticide class	Target site	Gene name	Contig Number	Coverage
Organophosphates, Carbamates	Acetylcholinesterase (AChE)	AChE 1	24052	1.59
		AChE 2	19680	1.63

neonicotinoids	Nicotinic acetylcholine receptor (nAChR)	nAChR alpha 2 subunit	19430 21473	2.87 1.66
		nAChR alpha 3 subunit	20111	3.16
		nAChR alpha 4 subunit	16361	2.47
		nAChR alpha 5 subunit	22076 35554 21985	2.01 1.33 1.70
		nAChR alpha 6 subunit	20921 29179 22598	1.89 3.37 1.46
		nAChR alpha 7 subunit	12555	3.50
		nAChR alpha 10 subunit	1918	15.0
		nAChR beta 1 subunit	31493 36485	1.55 1.37

Tetronic & Tetramic acid derivatives	Acetyl-CoA carboxylase (ACCase)	ACCase	28490 41433 17359 1349	1.84 1.18 1.84 10.7

Pyrethroids, Pyrethrins	Voltage-gated sodium channel (VGSC)	VGSC	22691 37637 21272	3.98 1.96 2.65

Organochlorines, Phenylpyrazoles (Fiproles)	GABA receptor	GABA receptor	16203 37638	4.37 1.47
	Glutamate-gated chloride channel (GluCl)	GluCl	35107 15229 35534	1.36 2.62 1.69

Diamides (chlorantraniliprole, cyanthraniliprole, flubendiamide)	Ryanodine receptor (RyR)	RyR	35833 7799	1.09 6.30

Additional file 7 includes their nucleotide sequences.

## Conclusions

*T. vaporariorum *is an important agricultural pest that has developed resistance to several insecticides used for whitefly control. To date, the lack of genomics data available for this species has hampered characterisation of the molecular mechanisms underlying resistance. The ~55,000 non-redundant EST contigs described in this study represent a dramatic expansion of existing cDNA sequence available for *T. vaporariorum*. We have identified the genes and gene families that are potential candidates for conferring insecticide resistance in *T. vaporariorum *including those encoding enzymes putatively involved in metabolic detoxification of xenobiotics and those encoding the target proteins of the major chemical classes of insecticides. The EST contig library developed in this study can be used as a reference transcriptome for analysis of gene expression using cDNA microarray and/or SAGE. We plan to use these genomic resources to investigate the role of detoxifying enzymes and target-site modification in *T. vaporariorum *populations that are resistant to insecticides. However, more broadly the annotated EST library will facilitate the investigation of the fundamental biology of *T. vaporariorum *and its interactions with host plants. *T. vaporariorum *has a similar biology to *B. tabaci*, offering the prospect of sharing information on resistance mechanisms and other biological traits between these major crop pests.

## Methods

### Insects and RNA extraction

Whiteflies for the generation of cDNA libraries were obtained from two different strains of *T. vaporariorum*. One was an insecticide susceptible standard strain (TV1) and the other was a strain from Turkey (TV6) selected with a 1,000 ppm dose of the neonicotinoid insecticide, imidacloprid (Confidor; Bayer CropScience). TV6 was collected from a greenhouse with a history of intensive insecticide use, although the complete treatment history is unknown for this strain. Insects were maintained on French bean plants, *Phaseolus vulgaris *L., cv. 'Canadian Wonder' (Fabaceae), under a 16h photoperiod at 24°C. More than 2,000 adults of each strain were collected in two separate 2 ml Eppendorf tubes and flash frozen in liquid nitrogen. Samples were sent to the University of Exeter (Cornwall Campus, Penryn, UK) in dry ice and stored at -80°C prior to RNA extraction.

RNA was isolated using TRIzol reagent (Invitrogen) according to the manufacturer's protocol. Genomic DNA contamination was removed by DNAse treatment (TURBO DNAse, Ambion) for 30 min at 37°C, RNA was further purified (RNeasy MinElute Clean up Kit, Qiagen) following the manufacturer's protocol and eluted in 20 μl of RNA storage solution (Ambion).

### cDNA library preparation, sequence pre-processing and assembly

Two cDNA libraries were used in order to identify as many genes encoding detoxification enzymes as possible. This may have been influenced by differences in gene expression levels in the two libraries, despite the fact that both libraries were normalised. Another reason for the use of the two cDNA libraries was to look for potential SNPs in target-site genes associated with insecticide resistance. Full-length, enriched, cDNAs were generated from 2 μg total RNA (SMART PCR cDNA synthesis kit, BD Clontech) following the manufacturer's protocol. Reverse transcription was performed using the PrimeScript reverse transcription enzyme (Takara) for 60 min at 42°C and 90 min at 50°C. In order to reduce over-abundant transcripts, double-stranded cDNAs were normalised using the Kamchatka crab duplex-specific nuclease method (Trimmer cDNA normalisation kit, Evrogen) [30]. Two aliquots, one of each of the normalised cDNA libraries, were 454 sequenced at the Advanced Genomics facility at the University of Liverpool. The two cDNA libraries were tagged prior to sequencing using molecular barcodes (Multiplex Identifiers, Roche Applied Sciences). A single full plate run (using both the TV1 and TV6 cDNA tagged libraries) was performed on the 454 GS-FLX Titanium series pyrosequencer (Roche Applied Science) using 3 μg of normalised cDNAs processed by the "shotgun" method. For raw reads pre-processing (removal of Poly-A tails and SMART adapters) and assembly, the custom pipeline est2assembly was used [12]. A pool of the processed reads from both cDNA libraries (TV1 and TV6) were clustered using the MIRA v2.9.26x3 assembler with the "*de novo*, normal, EST, 454" parameters, specifying a minimum read length of 40 nt, a minimum sequence overlap of 40 nt, and a minimum percentage overlap identity of 80%.

### Blast homology searches and sequence annotation

Blast homology searches and sequence annotations were carried out following a method that was successfully used for a midgut transcriptome of the tomato hornworm, *Manduca sexta *Linnaeus (Lepidoptera: Sphingidae) [8]. BLAST2GO software v.2.3.1 (http://www.blast2go.org) was used to perform several analyses of the EST assembly (contigs) [31]. Initially, homology searches were performed remotely on the NCBI server through QBLAST in a sequential strategy. Firstly, contig sequences were searched via BLASTx against the NCBI non-redundant (nr) database, using an E-value cut-off of 1E^-3 ^and selecting predicted polypeptides of a minimum length of 10 amino acids. Secondly, the sequences that did not receive any BLASTx hit were searched via BLASTn against the NCBI nr nucleotide database using an E-value cut-off of 1E^-10^. Also, BLASTx searches with an E-value cut-off of 1E^-5 ^were performed against the *D. melanogaster *uniprot (100) database. For gene ontology mapping (GO; http://www.geneontology.org), the program extracts the GO terms associated with homologies identified with NCBI's QBLAST and returns a list of GO annotations represented as hierarchical categories of increasing specificity. BLAST2GO allows the selection of a significance level for the false discovery rate, here used at a 0.05% probability level cut-off. GO terms were modulated using the annotation augmentation tool ANNEX [32], followed by GOSlim. GOSlim consists of a subset of the GO vocabulary encompassing key ontological terms and a mapping function between the full GO and the GOSlim. Here, we used the 'generic' GOSlim mapping term (goslim_generic.obo) available in BLAST2GO. Enzyme classification (EC) codes, and KEGG (Kyoto Encyclopedia of Genes and Genomes) metabolic pathway annotations, were generated from the direct mapping of GO terms to their enzyme code equivalents. Finally, InterPro (InterProScan, EBI) searches were performed remotely from BLAST2GO via the InterPro EBI web server. Potential ORFs (open reading frames) were identified using the ORF-predictor server (http://proteomics.ysu.edu/tools/OrfPredictor.html) [33]. An ORF cut-off of 200 bp was used.

### Manual curation of genes of interest, phylogenetic analysis and SNP identification

Contigs that had a protein motif of a cytochrome P450 or a protein domain of a CCE or a GST, as well as contigs that corresponded to the target sites of the most important chemical classes of insecticides were searched by BLASTn against all the assembled processed reads (http://www.rfc.ex.ac.uk/iceblast/iceblast.php) using an E-value cut-off of 1E^-4^. Each contig was reassembled from the reads that returned a BLAST hit and manually curated using Geneious software v.4.8.5 (Biomatters Ltd, Auckland, New Zealand), to check for potential frame-shifts and SNPs. Nucleotide sequences were dynamic translated using the EXPASY Proteomics Server (http://www.expasy.ch/tools/dna.html, Swiss Institute of Bioinformatics). All the identified sequences were searched by BLASTx against all the assembled contigs in the iceblast server using an E-value cut-off of 1E^-4 ^and the results with more than 99% similarity with the query sequence were eliminated as allelic variants (note that from those sequences, only the longest contigs with the best coverage were manually curated). MEGA 4.0 software [34] was used to perform multiple sequence alignment of P450s, CCEs, GSTs and nAChRs prior to phylogenetic analysis and to construct consensus phylogenetic trees using the neighbour-joining method. Bootstrap analysis of 1,000 replication trees was performed in order to evaluate the branch strength of each tree. The manually curated re-assembled contigs that encoded an insecticide target were investigated for the presence of SNPs arising due to nucleotide divergence between the two strains.

### Sequence submission

The raw nucleotide reads obtained by 454 sequencing were submitted to the Sequence Read Archive (SRA) database at NCBI with accession number SRA024353.1. An assembly of the *T. vaporariorum *data as well as the unassembled reads was uploaded to the InsectaCentral database (http://www.insectacentral.org/) and is searchable by BLAST at the following URL: http://www.rfc.ex.ac.uk/iceblast/iceblast.php. InsectaCentral is a central repository of insect transcriptomes, similar to the ButterflyBase, produced using traditional capillary sequencing or 454 pyrosequencing (NGS) [35]. Note that the names of the validated enzymes (see additional files 5-8) are made from the letters Tv followed by the number of the contig from the InsectaCentral database (For example IC88556AaEcon23678 is called Tv23678).

## Authors' contributions

ID, RFC, MW, KG and NK conceived the study. YP generated the cDNA libraries. YP and PW conducted preliminary data curation and transcriptome assembly. NK, RC and YP annotated, and manually curated genes of interest. DN named the manually curated P450 genes. NK and CB analysed the data (gene homology searches and annotation) using BLAST2GO software. NK and CB constructed the initial manuscript and all authors contributed to the preparation of the final version.

## Supplementary Material

Additional file 1 **Characteristics of assembled *Trialeurodes vaporariorum *454 contigs and BLASTx alignments against *Drosophila melanogaster***. (A,B) length and coverage of contigs, (C,D) percent identity and deduced amino acid alignment length for all blast hits to *D. melanogaster *predicted proteins (additional file 1.pdf) Click here for file

Additional file 2 **Top BLAST hits in the NCBI nr database for each unique contig**. Note that only the contigs that returned a BLAST result are shown in this file (additional file 2.xls) Click here for file

Additional file 3 E-value (A) and percentage similarity (B) distributions of the top BLAST hit for each contig of *Trialeurodes vaporariorum *(additional file 3.tiff) Click here for file

Additional file 4 Names, corresponding contig numbers, amino-acid sequences and *Acyrthosiphon pisum *or *Bemisia tabaci *orthologues of contigs that encode detoxifying enzymes (additional file 4.xls) Click here for file

Additional file 5 P450s nucleotide sequences (additional file 5.txt) Click here for file

Additional file 6 CCEs nucleotide sequences (additional file 6.txt) Click here for file

Additional file 7 GSTs nucleotide sequences (additional file 7.txt) Click here for file

Additional file 8 Nucleotide sequences of target sites of the most important insecticide classes (additional file 8.txt) Click here for file

Additional file 9 Single nucleotide polymorphisms (SNPs) arising due to nucleotide divergence between the two strains (TV1 and TV6) (additional file 9.xls) Click here for file
