# Supplementary material for: Pyrosequencing the transcriptome of the greenhouse whitefly, Trialeurodes vaporariorum reveals multiple transcripts encoding insecticide targets and detoxifying enzymes
Source: BMC Genomics. 2011 Jan 24;12:56. doi: 10.1186/1471-2164-12-56 (PMC3036619; doi:10.1186/1471-2164-12-56)
Supplement: Additional file 1 — Characteristics of assembled Trialeurodes vaporariorum 454 contigs and BLASTx alignments against Drosophila melanogaster. (A,B) length and coverage of contigs, (C,D) percent identity and deduced amino acid alignment length for all blast hits to D. melanogaster predicted proteins (additional file 1.pdf) [file 1471-2164-12-56-S1.PDF]

Graphs for the *T. vaporariorum* 454 assembly

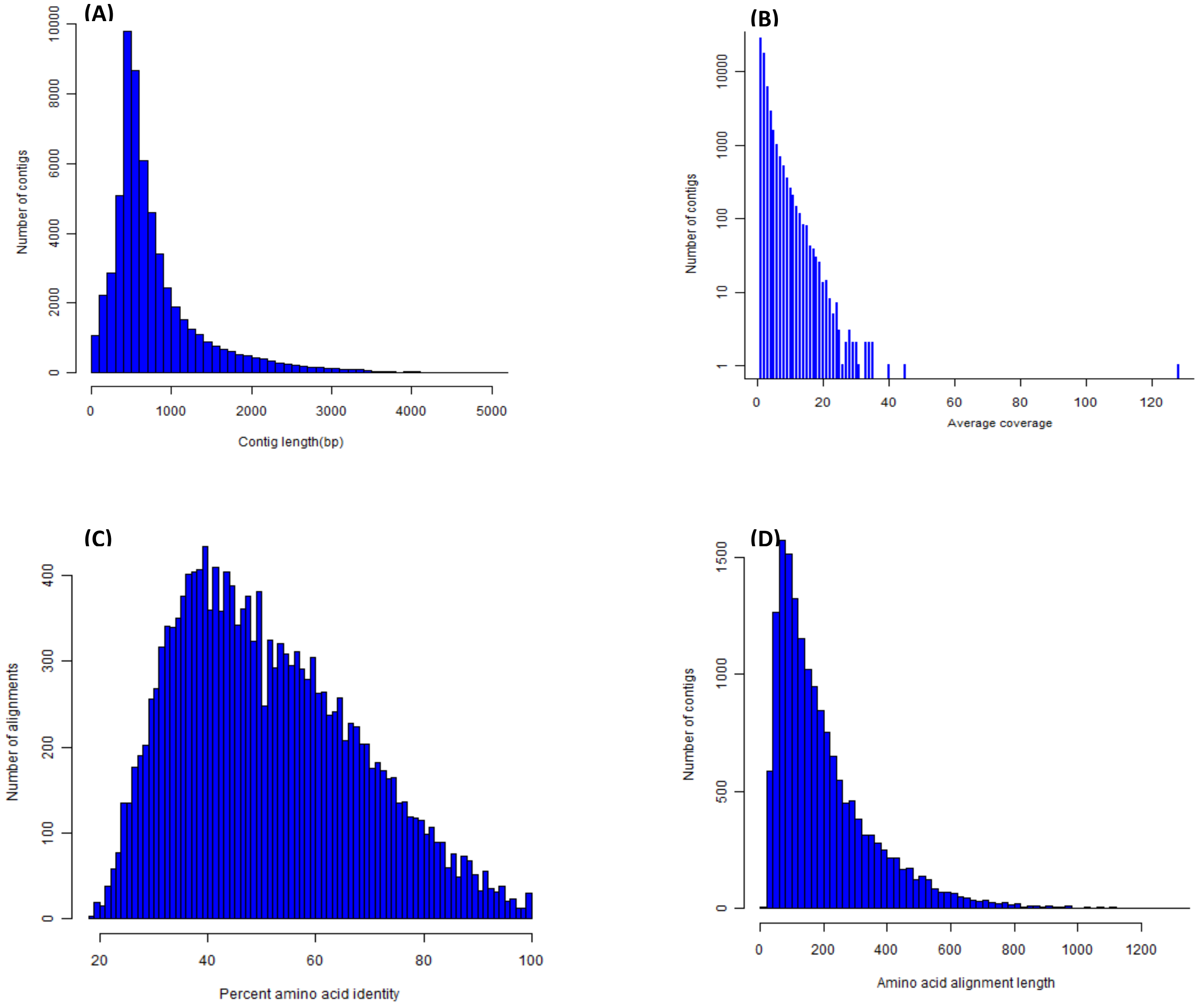

Characteristics of assembled *T. vaporariorum* 454 contigs and BLASTx alignments against *Drosophila melanogaster*. (A,B) length and coverage of contigs, (C,D) percent identity and deduced amino acid alignment length for all blast hits to *D.melanogaster* predicted proteins.
