# Supplementary figures and images for: Pyrosequencing the transcriptome of the greenhouse whitefly, Trialeurodes vaporariorum reveals multiple transcripts encoding insecticide targets and detoxifying enzymes
Source: BMC Genomics. 2011 Jan 24;12:56. doi: 10.1186/1471-2164-12-56 (PMC3036619; doi:10.1186/1471-2164-12-56)

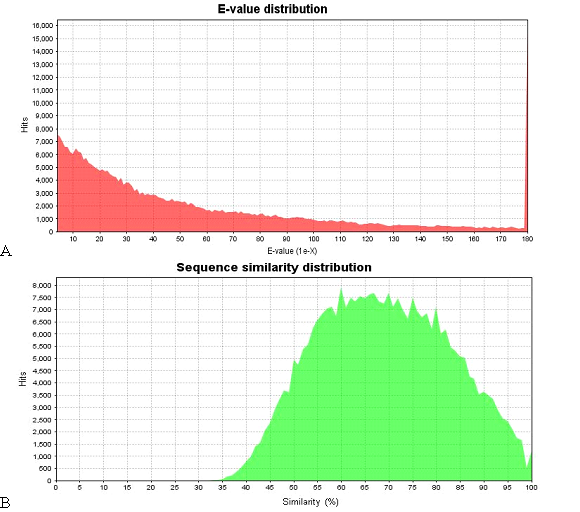

Supplement: Additional file 3 — E-value (A) and percentage similarity (B) distributions of the top BLAST hit for each contig of Trialeurodes vaporariorum (additional file 3.tiff) [file 1471-2164-12-56-S3.TIFF]
